# Opioids regulate the functional state of immune cells and reduce inflammatory cardiac injury: Role of opioid receptors, MRGPRX2, and TLR4

**DOI:** 10.7555/JBR.39.20250328

**Published:** 2026-03-19

**Authors:** Svetlana V. Gusakova, Maria Sirotina, Leonid N. Maslov, Alisa S. Slidnevskaya, Mikhail Kilin, Boris K. Kurbatov, Artur Kan, Ivan A. Derkachev, Feng Fu

**Affiliations:** 1 Laboratory of Experimental Cardiology, Cardiology Research Institute, Tomsk National Research Medical Center, Russian Academy of Sciences, Tomsk, Tomsk Oblast 634012, Russia; 2 Department of Physiology and Pathophysiology, Air Force Medical University, Xi'an, Shaanxi 710032, China

**Keywords:** heart, inflammatory injury, opioid receptors, MRGPRX2, TLR4

## Abstract

Neutrophils, macrophages, CD3^+^, CD4^+^, and CD8^+^ T lymphocytes express µ-, δ-, and κ-opioid receptors (ORs) with varying affinities for opioids. Mast cells express the atypical OR Mas-related G-protein-coupled receptor X2 (MRGPRX2), which has a low affinity for morphine. Neutrophils and macrophages can synthesize and release endogenous opioid peptides. Activation of ORs enhances the synthesis of proinflammatory cytokines and the production of reactive oxygen species (ROS) in unstimulated leukocytes. Conversely, OR activation reduces proinflammatory cytokine synthesis in stimulated neutrophils and macrophages. Morphine inhibits Toll-like receptor 4 (TLR4) expression in macrophages, thereby attenuating inflammation, whereas methadone induces ROS production in mast cells through TLR4 activation. Stimulation of TLR4 triggers β-endorphin synthesis in macrophages. The production of proinflammatory cytokines and ROS contributes to cardiac reperfusion injury. Importantly, activation of κ_1_- and µ-ORs suppresses proinflammatory cytokine production by leukocytes, thereby mitigating inflammatory injury to the heart and other organs.

## Introduction

Despite advancements in modern cardiology for treating acute myocardial infarction (AMI), the mortality rate associated with this condition remains high. In particular, patients with ST-segment elevation myocardial infarction (STEMI) and cardiogenic shock experience a notably high mortality rate^[1–2]^. When patients with STEMI are admitted within two hours of symptom onset, the in-hospital mortality rate is approximately 4%^[1]^. However, if admission occurs more than two hours after the onset of STEMI, the in-hospital mortality rate increases to 13%^[1]^. Cardiac reperfusion injury (CRI) and cardiogenic shock are among the primary causes of death in patients with AMI^[2–3]^. The in-hospital mortality rate for patients with Takotsubo syndrome (stress-induced cardiac injury [SICI]) ranges from 5%–7%^[4]^. Evidence suggests that proinflammatory cytokines, neutrophils, monocytes/macrophages, and CD4^+^ T-lymphocyte infiltration play a role in CRI^[3,5]^. Similarly, there is evidence that inflammation, cytokines, and macrophages are involved in the pathogenesis of Takotsubo syndrome^[6–9]^.

Opioid receptors (ORs) are G_i/o_ protein-coupled receptors^[10–11]^. The activation of ORs triggers an increase in intracellular cyclic guanosine monophosphate (cGMP), inositol-1,3,4-triphosphate, and diacylglycerol levels^[10–12]^. OR agonists inhibit cyclic adenosine monophosphate (cAMP) production when it is stimulated by forskolin or isoproterenol^[10–12]^. Evidence suggests that morphine and fentanyl reduce the activity of macrophages, neutrophils, natural killer cells, and T-cells^[13–14]^. In a murine line with a hyperactive opioid system, OR antagonists were administered for 12 days at a dose of 5 mg/(kg·day) *via* an osmotic minipump, and the percentage of T cells and T-cell subpopulations isolated from the spleen was measured^[15]^. Long-term administration of naloxone methiodide, a non-selective OR antagonist that does not cross the blood-brain barrier, modified the number of CD8^+^ and CD4^+^ cells; and long-term administration of the selective μ-OR antagonist cyprodime and the selective δ-OR antagonist naltrindole altered the number of CD69^+^ CD4^+^ cells and CD8^+^ cells^[15]^. These findings convincingly demonstrate that murine T cells preferentially express μ- and δ-ORs. Moreover, studies demonstrate that OR agonists can reduce CRI and SICI^[4,12,16–18]^; that the activation of peripheral µ_2_-, δ_2_-, and κ_1_-ORs alleviates CRI^[16,18–22]^; and that the activation of peripheral µ-OR increases cardiac tolerance to SICI^[17]^.

We hypothesize that the activation of µ_2_-, δ_2_-, and κ_1_-ORs in immune cells exerts an immunosuppressive effect, thereby enhancing cardiac tolerance to CRI and SICI. In this context, neutrophils, lymphocytes, and macrophages are expected to express µ-, δ-, and κ-ORs, and OR stimulation could decrease the functional activity of these cells. The purpose of this review is to summarize the role of opioid receptors in regulating the functional state of immune cells and the production of proinflammatory cytokines.

## The expression of ORs in immunocytes

### The expression of ORs, opioids, and the main effects of opioids in neutrophils

In 1995, µ-opioid receptor (*MOR*) mRNA was isolated from human and monkey neutrophils^[23]^. The expression of the µ-opioid receptor in human neutrophils was confirmed by Glattard *et al*^[24]^. Human neutrophils were found to synthesize and release Met-enkephalin and β-endorphin^[25]^. These opioid peptides produce a local antinociceptive effect in rats^[26]^. Human neutrophils have also been shown to synthesize and release endogenous morphine^[24]^. β-endorphin, Met-enkephalin, and dynorphin A have been found in human neutrophils^[27]^. The precursor of Met-enkephalin and β-endorphin pro-opiomelanocortin was detected in murine and human neutrophils^[27–28]^.

It was discovered that the non-selective OR agonist β-endorphin stimulated the production of superoxide radicals (O_2_^•−^) by human neutrophils^[29]^. The peak effect was observed at a final concentration of 1 pmol/L, with effects beginning at a concentration of 10 fmol/L. β-Endorphin exhibited a high affinity for µ_2_-OR, with a *K*_i_ ranging from 1.00 to 3.22 nmol/L, and δ-OR, with a *K*_i_ of 1 nmol/L^[30–31]^, but a low affinity for κ-OR, with a *K*_i_ greater than 1 μmol/L^[31]^. It is therefore expected that the peak response of O_2_^•−^ production would occur at a final concentration of 10–32 nmol/L. At a concentration of 10 fmol/L, β-endorphin did not affect ORs in the brain^[30–31]^. Naloxone (1 pmol/L) completely abolished β-endorphin-induced O_2_^•−^ generation^[29]^. The low-affinity OR agonist N-acetyl-β-endorphin had no effect on O_2_^•−^ production^[29]^. These findings indicate that neutrophils possess ORs with very high affinity for β-endorphin. It was also found that D-Ala^2^-methionine enkephalinamide (DAMEA) inhibited free radical generation by human neutrophils starting at a concentration of 1 fmol/L^[32]^. Naloxone (100 nmol/L) partially reversed the DAMEA-induced inhibition of free radical production^[32]^. The preferential µ-OR agonist morphine inhibited interleukin-8-induced migration of monkey neutrophils starting at a concentration of 1 pmol/L^[33]^. It should be noted that all opioids have *K*_i_ values in the nanomolar range^[30–31,34–36]^. Thus, it remains puzzling why β-endorphin and DAMEA exert their effects in the femtomolar range, whereas morphine acts at a concentration of 1 pmol/L, and why these agents exhibit opposite effects on free radical generation.

Morphine suppressed the expression of complement and surface receptors (CD11b, CD16, and CD35) in human neutrophils, starting at a concentration of 50 nmol/L, through µ_3_-OR stimulation^[37–38]^. Endomorphin-1 and endomorphin-2 inhibited the production of O_2_^•−^ induced by phorbol 12-myristate 13-acetate (PMA) in rat peritoneal neutrophils^[39]^. This inhibition was observed at a concentration as low as 0.1 fmol/L, peaking at 10 nmol/L. Conversely, endomorphin-1 and endomorphin-2 induced O_2_^•−^ production in unstimulated neutrophils at concentrations starting from 10 fmol/L^[39]^. The µ-OR antagonist β-funaltrexamine nullified the effects of endomorphins, while the selective δ-OR antagonist naltrindole did not. Hence, endomorphins modulate O_2_^•−^ generation in neutrophils *via* µ-OR activation. Endomorphin-1 and endomorphin-2 have *K*_i_ values for the µ-OR of 0.94 nmol/L and 1.57 nmol/L, respectively^[30]^. Consequently, it remains unclear why endomorphins exert effects within the femtomolar range.

The selective κ_1_-OR agonist U-50488 inhibited the chemotaxis of murine bone marrow neutrophils at a final concentration of 0.1 nmol/L^[40]^. The κ_2_-OR agonist bremazocine, the selective µ-OR agonist DAMGO, and the selective δ_1_-OR agonist DPDPE also suppressed neutrophil chemotaxis. Naloxone counteracted the effect of U-50488^[40]^. These findings indirectly indicate that neutrophils express κ_1_-, κ_2_-, µ-, and δ_1_-ORs. U-50488 has a *K*_i_ value of 0.12 nmol/L in cloned mouse ORs^[31]^. Therefore, the κ_1_-OR in neutrophils is identical to the κ_1_-OR expressed in other tissues. The effect of Met-enkephalin and β-endorphin on ROS production by unstimulated human neutrophils was also studied^[41]^. Met-enkephalin and β-endorphin at a final concentration of 10 nmol/L increased ROS generation by unstimulated neutrophils. Menzebach *et al*^[42]^ demonstrated that Met-enkephalin (1 nmol/L), β-endorphin (1 nmol/L), the δ-OR agonist DADLE (10 pmol/L), and the selective δ_1_-OR agonist DPDPE (10 pmol/L) triggered ROS production by unstimulated human neutrophils. However, naloxone and the selective δ-OR antagonist naltrindole abolished this effect of opioid peptides. The selective µ-OR antagonist CTOP had no impact on opioid-induced ROS generation^[42]^. Thus, the activation of δ_1_-OR induces an oxidative burst in neutrophils. Moreover, morphine (0.1–10 mg/kg) dose-dependently suppressed the infiltration of neutrophils into the peri-incisional tissue in mice^[43]^. Morphine (50 nmol/L) induced CD14 expression in human neutrophils^[44]^. Naloxone and Nω-nitro-L-arginine, a nitric oxide synthase (NOS) inhibitor, reversed morphine-induced CD14 expression; thus, investigators concluded that morphine stimulated CD14 expression through the activation of µ-OR and NOS in neutrophils^[44]^. Furthermore, β-endorphin (100 nmol/L) enhanced interleukin-1β production and inhibited interleukin-8 release by lipopolysaccharide (LPS)-stimulated human neutrophils^[45]^. Similarly, both β-endorphin (100 nmol/L) and the selective µ-OR agonist DAMGO (10 nmol/L) increased interferon-γ production by LPS-stimulated human neutrophils^[45]^.

**Figure 1:**
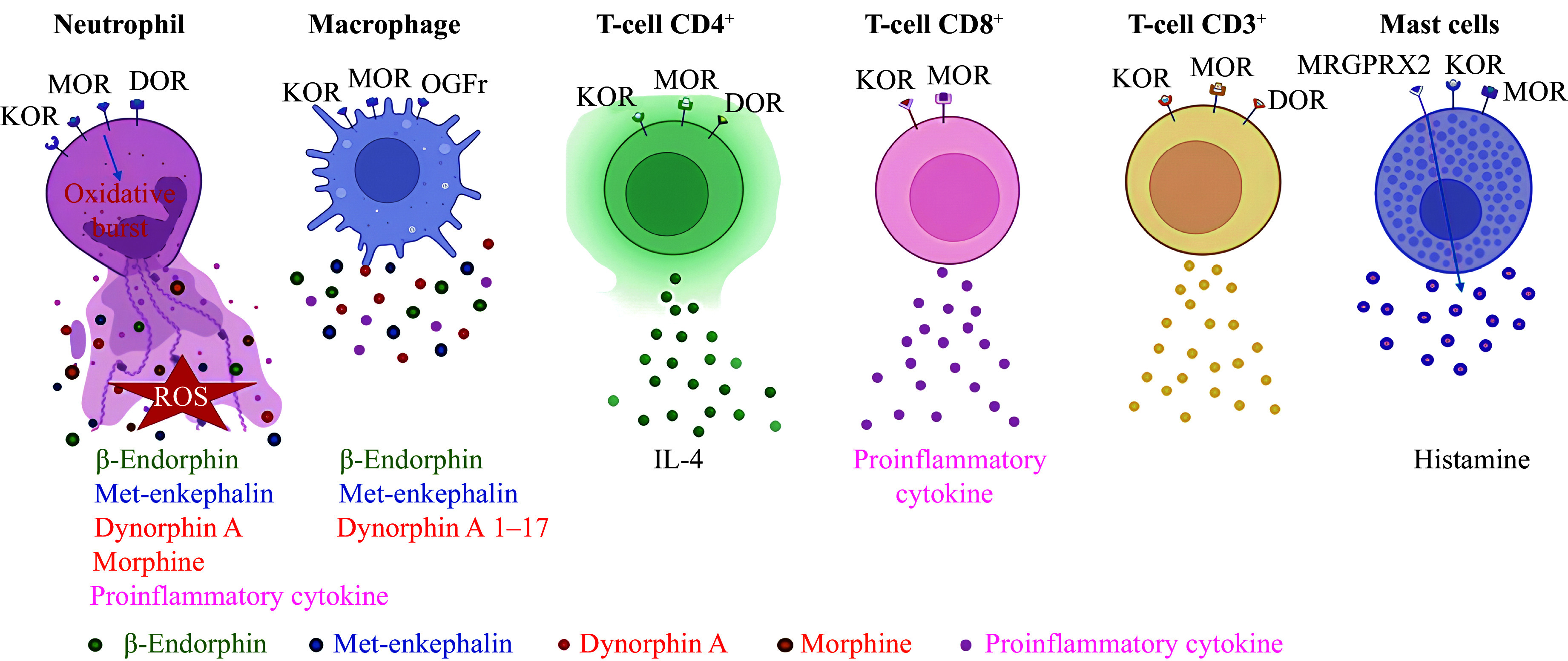
Opioid receptor expression and immune mediator release in neutrophils, macrophages, T cells, and mast cells. Neutrophils express MOR, KOR, and DOR, synthesize and release β-endorphin, Met-enkephalin, dynorphin A, and morphine; OR agonists induce oxidative burst in neutrophils. Macrophages express MOR, KOR, and OGFr, synthesize and release β-endorphin, Met-enkephalin, and dynorphin A 1–17. CD4^+^ T lymphocytes express MOR, DOR, and KOR, exhibiting both high-affinity and low-affinity ORs. Opioids increase IL-4 production in PHA-stimulated human CD4^+^ cells. CD8^+^ T lymphocytes express MOR and KOR, which have a high affinity for opioid peptides. Opioids increase proinflammatory cytokine production in unstimulated CD8^+^ T cells. CD3^+^ T lymphocytes express MOR, DOR, and KOR. Mast cells reportedly express KOR and MOR with low affinity for opioids; morphine induces histamine release through activation of the atypical opioid receptor MRGPRX2. Abbreviations: MOR, µ-opioid receptor; KOR, κ-opioid receptor; DOR, δ-opioid receptor; TLR4, Toll-like receptor 4; ROS, reactive oxygen species; IL-4, interleukin-4; OGFr, opioid growth factor receptor; MRGPRX2, Mas-related G-protein-coupled receptor X2.

In summary, evidence indicates that ORs expressed by neutrophils exhibit a higher affinity for opioid peptides^[29,32–33,39–40]^, compared with ORs found in the brain^[30–31,34–36]^ (***Table 1***). The expression of ORs appears to be context-dependent and varies across studies, suggesting that factors such as species, cell source, and stimulation conditions may affect their detectability. Opioids interact with ORs on neutrophils at concentrations in the femtomolar or picomolar range^[29,32–33,39–40]^ (***Tables 1*** and ***2***). In contrast, opioids interact with ORs in the brain at concentrations in the nanomolar range^[30–31,34–36]^. The reasons for these differences are not yet clear. OR agonists themselves induce an oxidative burst in neutrophils but inhibit the production of O_2_^•^^−^ by PMA-stimulated neutrophils. Opioids also inhibit the chemotactic activity of neutrophils. Additionally, there is evidence that neutrophils express MOR (***Table 1*** and ***Fig. 1***) and synthesize and release Met-enkephalin, β-endorphin, dynorphin A, and morphine (***Table 1*** and ***Fig. 1***).

**Table 1:** Opioid receptors of neutrophils, macrophages, and monocytes

Cells	Opioid	Effective concentration	OR affinity	OR subtype	Reference
Neutrophils	β-Endorphin	10 fmol/L	High	Unknown OR	[29]
Neutrophils	DAMEA	1 fmol/L	High	Unknown OR	[32]
Neutrophils	Morphine	1 pmol/L	High	Unknown OR	[33]
Neutrophils	Endomorphin-1, endomorphin-2	0.1 fmol/L	High	µ-OR	[39]
Neutrophils	DADLE, DPDPE	10 pmol/L	High	δ-OR, δ1-OR	[37]
Macrophages	U-50488	10 pmol/L	High	κ_1_-OR	[53]
Macrophages	Morphine	1 nmol/L	High	Unknown OR	[55]
Neutrophils	U-50488	0.1 nmol/L	Low	κ_1_-OR	[40]
Neutrophils	Met-enkephalin, β-endorphin	10 nmol/L	Low	Unknown R	[41]
Neutrophils	Met-enkephalin, β-endorphin	1 nmol/L	Low	δ-OR	[42]
Neutrophils	Morphine	50 nmol/L	Low	Unknown OR	[44]
Neutrophils	DAMGO, β-endorphin	100 nmol/L	Low	Unknown R	[45]
Macrophages	U-50488	4 µmol/L	Low	Unknown R	[51]
Macrophages	Morphine	100 nmol/L	Low	Unknown R	[54]
Macrophages	Morphine	300 µmol/L	Low	TLR4	[57]
Neutrophils	–	–	–	MOR	[23]
Neutrophils	–	–	–	µ-OR	[24]
Monocytes	–	–	–	µ-OR	[46]
Monocytes	–	–	–	MOR	[23]
Macrophages	–	–	–	MOR	[48]
Macrophages	–	–	–	KOR	[49]
Macrophages	–	–	–	MOR	[50]
Macrophages	–	–	–	KOR	[51]
Abbreviations: DADLE, Tyr-D-Ala-Gly-Phe-D-Leu-OH; DAMEA, D-Ala^2^-methionine enkephalinamide; DPDPE, Tyr-c[D-Pen-Gly-Phe-D-Pen]-OH; DAMGO, H-Tyr-D-Ala-Gly-Nα-Me-Phe-Gly-ol; KOR, κ-opioid receptor; MOR, µ-opioid receptor; OR, opioid receptor; R, receptor; TLR4, Toll-like receptor 4.					

### The expression of ORs, opioids, and the main effects of opioids in macrophages

In 1995, it was discovered that rat and monkey monocytes express µ-OR^[23,46]^. Evidence suggests that rat macrophages synthesize β-endorphin^[47]^ and that macrophage-like TPA-HL-60 cells express *MOR* mRNA^[48]^. J774 murine macrophages express κ-opioid receptor (*KOR*) mRNA and protein^[49]^. Interferon-γ stimulates KOR expression in these cells^[49]^. Human macrophages (CD68^+^ cells) also express MOR^[50]^, while the RAW264.7 macrophages express KOR^[51]^. Interleukin-4 induces the release of β-endorphin, Met-enkephalin, and dynorphin A 1–17 from murine proinflammatory M1 macrophages^[52]^ (***Table 1*** and ***Fig. 1***).

In LPS-stimulated rat NR8383 macrophages, the selective κ_1_-OR agonist U-50488 (10 pmol/L) inhibited the production of tumor necrosis factor-α (TNF-α) and interleukin-1β (IL-1β); pretreatment with the κ-OR antagonist nor-binaltorphimine (30 nmol/L) completely abolished this effect^[53]^. Moreover, U-50488 (4 µmol/L) suppressed proinflammatory cytokine (TNF-α and IL-6) expression in LPS-stimulated RAW264.7 cells^[51]^. Additionally, U-50488 inhibited LPS-induced proinflammatory M1 polarization of RAW264.7 macrophages^[51]^.

The preferential µ-OR agonist morphine at 100 nmol/L increased phagocytosis and upregulated ROS production in unstimulated human macrophages^[54]^. Morphine inhibited PMA-stimulated phagocytic activity of THP-1-derived macrophages with a half maximal inhibitory concentration (IC_50_) of 1 nmol/L^[55]^. This inhibitory effect peaked at 10 nmol/L morphine and was reversed by naloxone (10 µmol/L)^[55]^. Morphine has a *K*_i_ for the µ-OR of 4.6 nmol/L in brain membranes^[36]^. Thus, the macrophage µ-OR has a higher affinity for morphine than the µ-OR in the brain. This situation is analogous to that of neutrophil ORs, which also exhibit a higher affinity for opioids than the µ-OR in the brain. Hydromorphone (0.3 mg/kg intraperitoneally) reversed cardiopulmonary bypass-induced lung injury and suppressed pyroptosis of alveolar macrophages in rats^[56]^. It was observed that nuclear factor erythroid 2-related factor 2/heme oxygenase 1 (Nrf2/HO-1) played a role in the anti-pyroptotic effect of hydromorphone^[56]^. Incubation of the unstimulated RAW264.7 macrophages with morphine (300 µmol/L) for 6 or 24 h induced IL-6 and TNF-α expression and also resulted in mitochondrial ROS production^[57]^. These effects were negated by the Toll-like receptor 4 (TLR4) antagonist TAK242. Researchers employed a very high concentration of morphine (300 µmol/L), sufficient to stimulate all OR subtypes^[36]^, and did not use OR antagonists. Consequently, it remains unclear which OR is responsible for the effects of morphine. The influence of morphine on TLR4 expression was not studied. Therefore, it remains undetermined whether morphine directly stimulates TLR4 or modulates TLR4 expression.

There is indirect evidence suggesting that Met-enkephalin modifies the functional state of M2 macrophages by activating the opioid growth factor receptor (OGFr) expressed on both murine and human macrophages and monocytes^[58–60]^. The OGFr agonist, Met-enkephalin, has been shown to induce proinflammatory M1 polarization of macrophages in mice^[60]^.

These findings indicate that macrophages express MOR, KOR, and OGFr and synthesize and release β-endorphin, Met-enkephalin, and dynorphin A 1–17 (***Table 1*** and ***Fig. 1***). The activation of the κ_1_-OR suppresses proinflammatory cytokine release by LPS-stimulated macrophages, whereas OR activation in unstimulated macrophages enhances proinflammatory cytokine release and ROS production *via* TLR4.

### The expression of ORs, opioids, and the main effects of opioids in CD4^+^ T lymphocytes

CD4^+^ T lymphocytes, also known as T-helper cells, play a role in CRI^[3]^. In 1995, it was discovered that monkey CD4^+^ T lymphocytes expressed *KOR* mRNA^[61]^. By 1998, evidence was obtained indicating that murine CD4^+^ T cells expressed KOR^[62]^, and that both monkey and human CD4^+^ T lymphocytes expressed MOR mRNA and protein^[23,63]^. The delta-opioid receptor (DOR) was detected in human CD4^+^ T lymphocytes by immunofluorescence^[64]^ (***Table 2***).

β-Endorphin increased interleukin-4 (IL-4) production in concanavalin A-stimulated murine CD4^+^ T lymphocytes starting at a concentration of 10 pmol/L^[65]^. This effect peaked at 1 nmol/L. The opioid peptides α- and β-endorphin, but not Met-enkephalin, activated IL-2, IL-4, and interferon-γ production in concanavalin-stimulated CD4^+^ cells starting at 1 pmol/L^[66]^. Surprisingly, naloxone (1 nmol/L) also stimulated IL-2 and IL-4 production in murine CD4^+^ cells^[66]^. Therefore, the specific receptors involved in this effect of endorphins remain unknown. Morphine (700 nmol/L) induced a 250% increase in the cAMP level in isolated murine CD4^+^ cells through the activation of pertussis toxin-sensitive G_i/o_ proteins^[67]^. This is surprising because opioids typically inhibit cAMP synthesis *via* the activation of pertussis toxin-sensitive G_i/o_ proteins^[68]^. Since investigators did not use an OR antagonist, it is unclear which OR is involved in morphine-induced cAMP synthesis in CD4^+^ cells. The selective δ-OR agonist D-Ala^2^-deltorphin Ⅰ and the selective δ_1_-OR agonist DPDPE inhibited the proliferation of murine CD4^+^ cells starting at 10 pmol/L^[69]^. This effect reached its maximum at a concentration of 100 nmol/L. The selective δ-OR antagonist naltrindole (1 pmol/L) abolished the antiproliferative effect of deltorphin Ⅰ^[69]^. These data indirectly demonstrate that CD4^+^ cells express the δ-OR with very high affinity for opioid peptides. Additionally, β-endorphin (10 nmol/L) and the selective OR agonist DADLE (100 nmol/L) increased IL-4 production in phytohemagglutinin (PHA)-stimulated human CD4^+^ cells^[70]^.

In conclusion, CD4^+^ T lymphocytes express MOR, DOR, and KOR, exhibiting both high-affinity and low-affinity opioid receptors (***Table 2*** and ***Fig. 1***). Opioids have been shown to enhance IL-4 production in PHA-stimulated human CD4^+^ cells (***Fig. 1***).

### The expression of ORs, opioids, and the main effects of opioids in CD8^+^ T lymphocytes

CD8^+^ T lymphocytes, also known as cytotoxic T cells, are implicated in adverse post-ischemic cardiac remodeling^[71]^. Both human and murine CD8^+^ T lymphocytes express MOR mRNA and protein^[63,72]^. In 1998, evidence was obtained indicating that murine CD8^+^ T cells also expressed KOR^[62]^ (***Table 2***). The selective δ-OR agonist D-Ala^2^-deltorphin Ⅰ inhibited the proliferation of murine CD8^+^ T cells starting at 100 fmol/L^[69]^. The OR agonist Met-enkephalin (1 pmol/L) stimulated the proliferation of CD8^+^ T cells, increased production of the proinflammatory Fas-ligand, and induced the secretion of interferon-γ by murine CD8^+^ T cells^[72]^. Pretreatment with naltrexone (30 nmol/L) abolished these effects of Met-enkephalin.

In summary, CD8^+^ T lymphocytes express MOR and KOR, which have a high affinity for opioid peptides (***Table 2*** and ***Fig. 1***). The activation of ORs leads to an increase in proinflammatory cytokine production in unstimulated CD8^+^ T cells (***Fig. 1***).

### The expression of ORs, opioids, and the main effects of opioids in CD3^+^ T lymphocytes

CD3^+^ T lymphocytes, which are mature T cells, play a role in CRI^[3]^. In 1998, evidence was found that both murine and human CD3^+^ T cells expressed KOR^[62]^, DOR^[73–74]^, and MOR^[75]^. It was reported that the human T leukemia cell clone Jurkat, which expresses CD3, also expresses δ-OR^[76]^. It was demonstrated that anti-CD3/28 monoclonal antibodies induced MOR expression in MOR gene-transfected primary human T lymphocytes and Jurkat T cells^[77]^ (***Table 2***).

The non-selective OR agonist Met-enkephalin increased the intracellular Ca^2+^ concentration starting at 10 pmol/L in the human CD3^+^ T-leukemia cell clone, Jurkat^[76]^. The preferential µ-OR agonist morphine, starting from 10 nmol/L, inhibited interferon-γ production by murine T cells stimulated with anti-CD3 antibody^[78]^. MOR knockout abolished this effect of morphine in mice^[78]^. Morphine (10 nmol/L) increased cAMP synthesis in CD3^+^ T cells. The G_i/o_-protein inhibitor pertussis toxin eliminated morphine-induced inhibition of interferon-γ production and cAMP synthesis^[78]^. This finding was surprising because opioids are generally known to inhibit cAMP synthesis through the activation of G_i/o_-proteins^[68]^. Morphine (1–100 µmol/L) induced phosphorylation of extracellular signal-regulated kinase (ERK) and inhibited phosphorylation of nuclear factor-κB (NF-κB) in PMA-stimulated human CD3^+^ T cells^[79]^. Naloxone did not abolish these effects of morphine. Thus, the receptors involved in these effects of morphine remain unidentified.

Therefore, CD3^+^ T lymphocytes express MOR, DOR, and KOR (***Table 2*** and ***Fig. 1***). Morphine suppresses interferon-γ production by activating MOR coupled with G_i/o_-protein and enhances cAMP synthesis in stimulated CD3^+^ T cells. Additionally, morphine inhibited NF-κB activation and induced ERK phosphorylation in PMA-pretreated CD3^+^ T cells.

**Table 2:** Opioid receptors of T cells

Cells	Opioid	Effective concentration	OR affinity	OR subtype	Reference
CD4^+^ T cells	β-Endorphin, α-Endorphin	1–10 pmol/L	High	Unknown R	[65]
CD4^+^ T cells	Deltorphin Ⅰ, DPDPE	10 pmol/L	High	δ-OR	[69]
CD3^+^ T cells	Met-enkephalin	10 pmol/L	High	Unknown R	[76]
CD8^+^ T cells	Deltorphin Ⅰ	10 fmol/L	High	Unknown R	[69]
CD8^+^ T cells	Met-enkephalin	1 pmol/L	High	Unknown R	[72]
CD4^+^ T cells	β-Endorphin, DADLE	10–100 nmol/L	Low	Unknown R	[45]
CD3^+^ T cells	Morphine	10 nmol/L	Low	µ-OR	[78]
CD3^+^ T cells	Morphine	100 µmol/L	Low	Unknown R	[79]
CD3^+^ T cells	–	–	–	KOR	[62]
CD3^+^ T cells	–	–	–	δ-OR	[76]
CD3^+^ T cells	–	–	–	DOR	[73]
CD3^+^ T cells	–	–	–	MOR	[78]
CD4^+^ T cells	–	–	–	KOR	[61]
CD4^+^ T cells	–	–	–	MOR	[23]
CD4^+^ T cells	–	–	–	MOR	[75]
CD4^+^ T cells	–	–	–	KOR	[62]
CD4^+^ T cells	–	–	–	MOR	[63]
CD4^+^ T cells	–	–	–	DOR	[64]
CD8^+^ T cells	–	–	–	KOR	[62]
CD8^+^ T cells	–	–	–	MOR	[63]
CD8^+^ T cells	–	–	–	MOR	[73]
Abbreviations: DADLE, Tyr-D-Ala-Gly-Phe-D-Leu-OH; DPDPE, Tyr-c[D-Pen-Gly-Phe-D-Pen]-OH; DOR, δ-opioid receptor; KOR, κ-opioid receptor; MOR, µ-opioid receptor; OR, opioid receptor; R, receptor.					

### The expression of ORs, opioids, and the main effects of opioids in mast cells

In 2024, Martínez-Cuevas *et al*^[80]^ detected µ-OR in murine mast cells using immunofluorescence. There is indirect evidence that mast cells express KOR in humans^[81]^ (***Table 3***).

**Table 3:** The opioid receptors of mast cells

Cells	Opioid	Effective concentration	OR affinity	OR subtype	Reference
Mast cells	Morphine	100 µmol/L	Low	Unknown R	[82]
Mast cells	Morphine	1 mmol/L	Low	TLR4?	[89]
Mast cells	Morphine	35 µmol/L	Low	MRGPRX2	[121]
Mast cells	Morphine	10 µmol/L	Low	MRGPRX2	[92]
Mast cells	Morphine	3.5 µmol/L	Low	Unknown R	[93]
Mast cells	Morphine	354 µmol/L	Low	Unknown R	[94]
Mast cells	Methadone	0.5 mmol/L	Low	MRGPRX2	[80]
Mast cells	–	–	–	µ-OR	[80]
Mast cells	–	–	–	KOR	[81]
Mast cells	–	–	–	MRGPRX2	[91]
Abbreviations: MRGPRX2, Mas-related G-protein-coupled receptor X2; OR, opioid receptor; R, receptor; TLR4, Toll-like receptor 4.					

Morphine, at concentrations ranging from 1 to 200 µmol/L, did not influence histamine release from human mast cells derived from pulmonary, intestinal, and cardiac tissues, nor from blood basophils^[82]^. However, morphine (at a concentration of 100 µmol/L) induced histamine release from human cutaneous mast cells^[82]^. The selective µ-OR agonist remifentanil, when administered intravenously at a dose of 30 µg/kg, decreased heart rate in humans but did not change plasma histamine levels^[83]^. It was observed that the subcutaneous injection of morphine and codeine triggered histamine release from human dermal mast cells^[84]^. Fentanyl, alfentanil, sufentanil, remifentanil, and buprenorphine did not provoke histamine release^[84]^. These findings suggest that the induction of histamine release by morphine or codeine is not dependent on OR activation. Neither the µ-OR agonist methadone nor the selective µ-OR agonist fentanyl affected plasma histamine levels in humans^[85]^. Intravenous administration of morphine (1 mg/kg, equivalent to 3.5 µmol/kg) resulted in a 2500-fold increase in plasma histamine levels compared with baseline in dogs^[86]^; moreover, morphine (at a dose of 0.5 mg/kg, equivalent to 1.8 µmol/kg) caused a 16-fold increase in plasma histamine levels from baseline; however, morphine did not affect heart rate and blood pressure in dogs^[86]^. It was reported that pretreatment with morphine at a dose of 0.3 mg/kg reduced infarct size in rats subjected to coronary artery occlusion (CAO) and reperfusion^[87]^. The maximum dose of morphine used in treating patients with AMI is 0.125 mg/kg^[88]^. Notably, researchers did not test morphine at these clinically relevant doses.

Morphine, at concentrations ranging from 100 to 1000 µmol/L, inhibited the secretion of TNF-α by LPS-stimulated murine bone marrow-derived mast cells by downregulating TLR4 expression^[89]^. It is plausible that morphine directly binds to TLR4, as this effect was observed at a high concentration of morphine (1000 µmol/L). At 35 µmol/L, morphine induced degranulation in human laboratory of allergic diseases 2 (LAD2) mast cells^[90]^. Investigators proposed that this degranulation effect could be mediated through activation of Mas-related G protein-coupled receptor member X2 (MRGPRX2), a receptor with low affinity for morphine that is not blocked by naloxone^[91]^. Consequently, this effect has been termed atypical OR activation. It was shown that LAD2 human mast cells expressed MRGPRX2, and that MRGPRX2 siRNA abolished morphine-induced degranulation in these cells. Remifentanil (120 µmol/L) did not induce degranulation in human mast cells^[90]^. Thus, high doses of morphine can trigger mast cell degranulation. Morphine, at concentrations of 10 to 100 µmol/L, induced histamine release from human skin mast cells but not from synovial or lung mast cells^[92]^. Investigators suggested that this selective response was due to MRGPRX2 being exclusively expressed in human skin mast cells. Morphine at 3.5 µmol/L did trigger histamine release from canine BR and C2 mast cells^[93]^. At 354 µmol/L, morphine induced degranulation in primary umbilical cord blood human mast cells^[94]^. Fentanyl had no effect at the tested concentrations (1 µmol/L and 10 µmol/L)^[94]^. Furthermore, it was demonstrated that morphine-induced mast cell degranulation in pigs was not sensitive to naloxone^[95]^.

In 2020, Chéret *et al*^[81]^ discovered that the κ-OR agonist KOR-5a (200 nmol/L) stimulated the proliferation of human dermal mast cells *in vitro*. However, the researchers did not employ OR antagonists, leaving it unclear whether the κ-OR is implicated in the proliferative effect of KOR 5a. Typically, OR agonists bind to ORs within the nanomolar range^[31]^. Consequently, it is plausible that dermal mast cells express KOR. The µ-OR agonist methadone (500 µmol/L) induced ROS-dependent death in murine bone marrow-derived mast cells^[80]^. Naloxone and the µ-OR antagonist β-funaltrexamine partially attenuated this cytotoxic effect of methadone. This suggests that mast cells may express µ-ORs with a low affinity for opioids.

Thus, morphine induces histamine release at high doses/concentrations by activating the atypical opioid receptor MRGPRX2 (***Table 3*** and ***Fig. 1***). It is quite evident that histamine release is one of the manifestations of the toxic effects of high doses/concentrations of morphine^[96]^. It is possible that mast cells express KOR and MOR with a low affinity for opioids (***Table 3***).

## The interaction between ORs and TLR4

The activation of TLR4 leads to inflammation and pyroptotic death of cardiomyocytes; thus, its stimulation triggers CRI and adverse remodeling of the heart^[19,97]^.

Evidence suggests that opioids can induce TLR4 expression^[98–102]^. Intrathecal injection of morphine-3-glucuronide resulted in hyperalgesia and increased TLR4 expression in the microglia of the rat spinal cord^[98]^. Morphine, at a concentration of 1 µmol/L, has been shown to upregulate TLR4 expression in lung cancer cells and to decrease cell viability^[99]^. Morphine, at a concentration of 100 nmol/L, increased the *TLR4* mRNA levels in B16 melanoma cells^[100]^. Chronic administration of morphine [10 mg/(kg·day) subcutaneously] for 7 days led to increased TLR4 expression in periaqueductal gray microglia in mice^[101]^. The selective µ-OR agonist fentanyl induced TLR4 expression in spinal cord neurons and glial cells one day after injection^[102]^. The µ-OR agonist methadone (500 µmol/L) induced ROS production by murine mast cells^[80]^. This effect was not observed in TLR4-deficient mast cells. Therefore, investigators proposed that methadone induced ROS production in mast cells at high concentrations through the activation of TLR4^[80]^.

In contrast to µ-OR agonists such as fentanyl and morphine, the selective κ-OR agonist U-50488 inhibited TLR4 expression in the ischemic area following CAO and reperfusion in rats^[103]^. Morphine (at a concentration of 100 µmol/L) also suppressed TLR4 expression in RAW 264.7 macrophages^[104]^. Naloxone reversed this effect of morphine. It should be noted that morphine at a final concentration of 100 µmol/L activates all ORs^[31]^. Consequently, it remains unclear which specific OR is involved in the inhibition of TLR4 expression. Morphine (at a dose of 20 mg/kg) also suppressed TLR4 expression in murine peritoneal macrophages. This effect was partially reversed by naloxone^[104]^. These data suggest that not only an unidentified OR but also a yet-unidentified receptor may be involved in morphine-induced suppression of TLR4 expression.

Furthermore, it has been demonstrated that the activation of TLR4 by LPS stimulates β-endorphin synthesis in rat macrophages^[47]^ and induces MOR expression^[48]^ in macrophage-like TPA-HL-60 cells.

**Figure 2:**
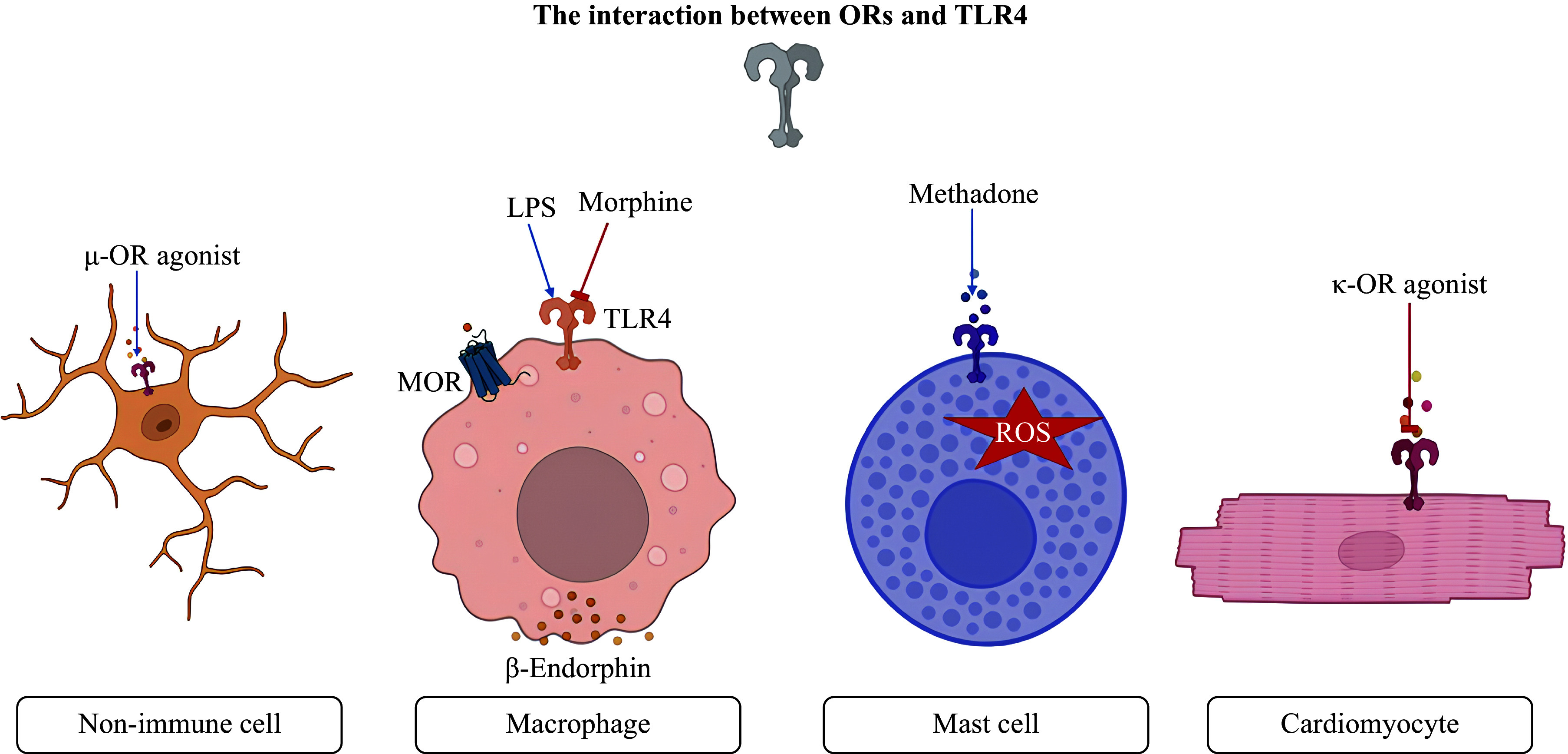
The µ-OR agonists stimulate TLR4 expression in non-immune cells. LPS induces TLR4 activation and stimulates β-endorphin synthesis and MOR expression in macrophages. Morphine suppresses TLR4 expression in macrophages. Methadone induces ROS production in murine mast cells *via* TLR4 activation. The κ-OR agonist U-50488 inhibits TLR4 expression in cardiomyocytes. Abbreviations: µ-OR, mu-opioid receptor; LPS, lipopolysaccharides; MOR, µ-opioid receptor; TLR4, Toll-like receptor 4; ROS, reactive oxygen species; KOR, κ-opioid receptor.

Hence, µ-OR agonists such as fentanyl and morphine can stimulate TLR4 expression in non-immune cells. Morphine suppresses TLR4 expression in macrophages. Methadone induces ROS production in murine mast cells through the activation of TLR4. The κ-OR agonist U-50488 inhibits TLR4 expression in cardiomyocytes. The TLR4 agonist LPS induces β-endorphin synthesis in macrophages (***Fig. 2***).

## Opioids reduce the inflammatory injury of the heart

In a rat model undergoing CAO for 30 min followed by reperfusion (180 min), the administration of the selective κ_1_-OR agonist U-50488 (1.5 mg/kg intravenously) 5 min before reperfusion reduced infarct size and neutrophil invasion in the ischemic area; however, the selective κ-OR antagonist nor-binaltorphimine abolished both effects of U-50488^[105]^. The authors suggested that limiting neutrophil invasion contributed to the reduction in infarct size. The same group of investigators confirmed the ability of U-50488 to reduce infarct size and attenuate neutrophil infiltration in the area at risk during reperfusion by inhibiting the expression of TLR4 and NF-κB, which are involved in inflammation^[103]^. U-50488 also reduced the adhesion of monocytes and neutrophils to endothelial cells and suppressed neutrophil migration in rats with salt-sensitive hypertension^[106]^.

It has been reported that the subcutaneous injection of the selective µ-OR agonist DAMGO (at a dose of 0.01 mg/kg) alleviated ischemia/reperfusion injury in the small intestine of mice and reduced neutrophil infiltration into the intestine^[107]^. The selective µ-OR antagonist CTAP counteracted this effect of DAMGO. Additionally, DAMGO decreased neutrophil infiltration in experimental colitis in mice^[108]^. It is plausible that DAMGO could reduce ischemia/reperfusion injury in the heart by activating the µ-OR, and thereby limiting neutrophil invasion. Experimental pancreatitis caused a threefold increase in serum creatine kinase-MB concentration, induced apoptosis of cardiomyocytes, and elevated myocardial levels of IL-1β and IL-6^[109]^. Pretreatment with the selective µ-OR agonist fentanyl (administered intravenously at a dose of 30 µg/kg) attenuated these changes^[109]^.

Myocardial injury is one of the most common complications of sepsis^[110]^. LPS induced a sepsis-like state in rats^[111]^. LPS caused the death of H9C2 cardiomyoblasts, increased lactate dehydrogenase release, and elevated malondialdehyde (MDA) production in H9C2 cells; LPS also activated protein kinase Cβ (PKCβ) and triggered autophagy. However, the µ-OR agonist remifentanil (2.5 µmol/L) reduced cell death, alleviated oxidative stress, decreased PKCβ activity, and inhibited autophagy in H9C2 cells^[112]^. It should be noted that remifentanil activated all opioid receptors at this concentration^[113]^. Therefore, it remains unknown which OR is involved in the cytoprotective effect of remifentanil. LPS triggered an increase in TNF-α, IL-6, and MDA content in lung and kidney tissues in rats^[111]^. Remifentanil reduced these alterations. Investigators did not use OR antagonists^[111]^. The µ-OR agonist oxycodone (50 µmol/L) protected H9C2 cells against LPS-induced injury, decreased creatine kinase-MB (CK-MB), troponin I, and MDA release from H9C2 cells, and inhibited ROS production^[110]^. Oxycodone reduced LPS-induced pyroptosis in H9C2 cells through an Nrf2-mediated increase in heme oxygenase (HO-1) expression. Oxycodone activated both µ-OR and κ-OR at a final concentration of 50 µmol/L^[114]^. Therefore, it remains unknown which OR is involved in the cytoprotective effect of oxycodone. Oxycodone mitigated LPS-induced myocardial dysfunction and reduced the serum CK-MB and IL-18 levels in wild-type mice but not in Nrf2-knockout mice^[110]^. Oxycodone also alleviated LPS-induced pyroptosis in myocardial tissue in wild-type mice but not in Nrf2-knockout mice^[110]^. Hence, µ-OR agonists reduced the inflammatory injury of the heart. However, the role of the µ-OR in this effect remains unexplored.

Activation of the β_1_-adrenergic receptor (β_1_-AR) plays a key role in SICI^[115]^; therefore, investigators use the β_1_- and β_2_-AR agonist isoproterenol to mimic SICI. Isoproterenol (85 mg/kg subcutaneously) induced severe contractile dysfunction and inflammatory injury in the rat heart^[116]^. The µ-OR agonist remifentanil (40 µg/kg daily, intraperitoneally) improved contractile function, reduced the serum CK-MB level and MDA content in myocardial tissue, and inhibited apoptosis of cardiomyocytes. Remifentanil decreased the serum levels of IL-6, TNF-α, and IL-1β. Remifentanil increased the expression of AMP-activated kinase (AMPK), which stimulates autophagy^[20]^. Autophagy is a reversible survival mechanism in its early stages that allows cells to endure stress^[20]^; therefore, AMPK activation could be considered a beneficial effect of remifentanil. Remifentanil suppressed isoproterenol-induced phosphorylation (activation) of C-Jun N-terminal kinase (JNK) in the rat heart^[116]^. Since JNK plays a triggering role in necroptosis^[117]^, its inhibition could be viewed as another beneficial effect of remifentanil. The specific OR mediating these effects remains unknown because OR antagonists were not used. However, given the low dose of remifentanil (40 µg/kg) used, it is plausible that µ-OR contributes to the cardioprotective and anti-inflammatory effects of remifentanil.

Therefore, current evidence suggests that the activation of κ_1_- and µ-ORs reduces inflammatory injury in the heart and other organs.

## Unresolved issues

It remains unclear why ORs expressed by neutrophils, macrophages, CD4^+^ T cells, CD3^+^ T cells, and CD8^+^ T cells exhibit a higher affinity for opioids than ORs found in the brain. Additionally, the reason behind the stimulation of cAMP synthesis upon the activation of ORs coupled with G_i/o_-proteins is also not well understood.

The chronic administration of OR antagonists, such as naloxone methiodide, cyprodime, and naltrindole, alters the functional state of T lymphocytes in mice *in vivo*^[15]^. Naloxone (at a concentration of 1 nmol/L) has been shown to stimulate the production of IL-2 and IL-4 in cultures of murine CD4^+^ cells *in vitro*^[66]^. It is conceivable that, in some instances, endogenous opioid peptides could stimulate ORs on T lymphocytes *in vivo*. Murine CD4^+^ cell cultures are capable of synthesizing α- and β-endorphins^[66]^, suggesting that these peptides may function as autacoids. In such cases, naloxone alone could modify IL-2 and IL-4 production. An alternative explanation for the effects of OR antagonists may be the spontaneous activation of G protein-coupled receptors in the absence of agonists^[118]^. Certain antagonists, known as inverse agonists, can interact with these receptors^[118]^. In contrast, neutral antagonists do not interact with these receptors but can compete with agonists for the receptor site. It has been determined that naltrindole acts as a neutral antagonist^[119]^, whereas cyprodime and naloxone are inverse agonists^[120–121]^. Consequently, the impact of OR antagonists on the functional state of T lymphocytes could result from competition with endogenous opioid peptides for OR binding or from the agonist-independent OR signaling. The resolution of these questions will likely be achieved through future studies.

## Conclusion

Neutrophils express µ-, κ_1_-, κ_2_-, and δ_1_-ORs. They synthesize Met-enkephalin, β-endorphin, dynorphin A, and morphine. Opioid receptors on neutrophils have a greater affinity for opioid peptides than those expressed by neurons. Opioids stimulate these neutrophil ORs at femtomolar and picomolar concentrations. They induce an oxidative burst in neutrophils but inhibit the production of O_2_^•^^−^ by PMA-stimulated neutrophils. Opioids also inhibit the chemotaxis of neutrophils. Opioid receptor agonists cause the release of proinflammatory cytokines and ROS production in unstimulated neutrophils.

Macrophages express MOR, KOR, and OGFr. These cells synthesize β-endorphin, Met-enkephalin, and dynorphin A 1–17. The activation of ORs in unstimulated macrophages promotes the release of proinflammatory cytokines and ROS. However, the activation of the κ_1_-OR reduces proinflammatory cytokine release from LPS-stimulated macrophages.

CD4^+^ T lymphocytes express MOR, DOR, and KOR, with both high- and low-affinity ORs. Opioid peptides increase IL-4 production in stimulated CD4^+^ cells. CD8^+^ T lymphocytes express MOR and KOR, with high-affinity ORs for opioid peptides. The activation of ORs promotes an increase in proinflammatory cytokine production in unstimulated CD8^+^ T cells. CD3^+^ T lymphocytes express MOR, DOR, and KOR. Morphine inhibits interferon-γ production through the activation of MOR in stimulated CD3^+^ cells. Morphine inhibits the activation of NF-κB and ERK in PMA-pretreated CD3^+^ T cells. Evidence suggests that morphine can increase cAMP synthesis through the activation of G_i/o_-proteins in CD3^+^ T cells and CD4^+^ cells.

There is evidence that mast cells express KOR and MOR with a low affinity for opioids. Morphine induces histamine release in large doses and at high concentrations through the activation of the atypical opioid receptor MRGPRX2. Histamine release is one of the manifestations of the toxic effects of large doses of morphine. Fentanyl and morphine cause TLR4 expression in non-immune cells. The κ-OR agonist U-50488 inhibits TLR4 expression in cardiomyocytes. Morphine inhibits TLR4 expression in macrophages. Methadone causes ROS production by mast cells through the activation of TLR4. The TLR4 agonist LPS induces β-endorphin synthesis in macrophages. The activation of κ_1_- and µ-ORs reduces proinflammatory cytokine production and inflammatory injury in the heart and other organs. Immune cells both express high-affinity ORs and synthesize opioid peptides, making them targets for opioid receptor agonists. Notably, these agonists can inhibit immune responses *via* OR activation in immune cells.
